# Heterologous vaccination with inactivated vaccine and mRNA vaccine augments antibodies against both spike and nucleocapsid proteins of SARS-CoV-2: a local study in Macao

**DOI:** 10.3389/fimmu.2023.1131985

**Published:** 2023-05-12

**Authors:** Hoi Man Ng, Chon Lok Lei, Siyi Fu, Enqin Li, Sek In Leong, Chu Iong Nip, Nga Man Choi, Kai Seng Lai, Xi Jun Tang, Chon Leng Lei, Ren-He Xu

**Affiliations:** ^1^ Laboratory Department, Kiang Wu Hospital, Macao, Macao SAR, China; ^2^ Faculty of Health Sciences, University of Macau, Macao, Macao SAR, China; ^3^ Laboratory Department, Zhuhai Hospital of Integrated Traditional Chinese and Western Medicine, Zhuhai, China

**Keywords:** SARS-CoV-2, inactivated vaccine, mRNA vaccine, heterologous vaccinations, antibodies, spike proteins, nucleocapsid proteins, Macao

## Abstract

The mRNA vaccines (RVs) can reduce the severity and mortality of severe acute respiratory syndrome coronavirus (SARS-CoV-2). However, almost only the inactivated vaccines (IVs) but no RVs had been used in mainland China until most recently, and the relaxing of its anti-pandemic strategies in December 2022 increased concerns about new outbreaks. In comparison, many of the citizens in Macao Special Administrative Region of China received three doses of IV (3IV) or RV (3RV), or 2 doses of IV plus one booster of RV (2IV+1RV). By the end of 2022, we recruited 147 participants with various vaccinations in Macao and detected antibodies (Abs) against the spike (S) protein and nucleocapsid (N) protein of the virus as well as neutralizing antibodies (NAb) in their serum. We observed that the level of anti-S Ab or NAb was similarly high with both 3RV and 2IV+1RV but lower with 3IV. In contrast, the level of anti-N Ab was the highest with 3IV like that in convalescents, intermediate with 2IV+1RV, and the lowest with 3RV. Whereas no significant differences in the basal levels of cytokines related to T-cell activation were observed among the various vaccination groups before and after the boosters. No vaccinees reported severe adverse events. Since Macao took one of the most stringent non-pharmaceutical interventions in the world, this study possesses much higher confidence in the vaccination results than many other studies from highly infected regions. Our findings suggest that the heterologous vaccination 2IV+1RV outperforms the homologous vaccinations 3IV and 3RV as it induces not only anti-S Ab (to the level as with 3RV) but also anti-N antibodies (via the IV). It combines the advantages of both RV (to block the viral entry) and IV (to also intervene the subsequent pathological processes such as intracellular viral replication and interference with the signal transduction and hence the biological functions of host cells).

## Introduction

The severe acute respiratory syndrome coronavirus (SARS-CoV-2) that causes the coronavirus disease 2019 (COVID-19) has evolved to an infectiously stronger but pathologically weaker variant Omicron than its previous ones (1–3). However, it remains crucial to generate new and effective vaccines against the ever-evolving derivatives of the virus to control or reduce the resurgences of the disease (4–8). For example, with the recent relaxation of the strictest and longest anti-pandemic strategies in China, it may raise concerns about the use of the limited types of vaccines for one of the largest populations in the world when it comes to tolerating Omicron infection (9). There are two major categories of vaccine development, *i.e.*, protein- and gene-based, against viral diseases. Protein-based vaccines are conventional vaccines that are generated through attenuation, inactivation, or recombination of whole or individual viral proteins, which are delivered as immunogens to activate the adaptive and humoral immune response. Gene-based vaccines are delivered via a DNA or RNA vector and are expressed in host cells to produce corresponding antigens which induce the immune response. Both protein- and gene-based vaccines have been employed for prevention of COVID-19 (10).

The immune response to SARS-CoV-2 vaccines includes the secretion of antibodies (Abs) by B cells, viral-specific CD4 and CD8 T cell responses, and antibody-dependent cell-mediated cytotoxicity (ADCC) by natural killer cells. Neutralizing antibodies (NAbs) are produced naturally by the body as part of the immune response to defend from pathogens, and their production is triggered by both infections and vaccinations against infections. The outer surface of SARS-CoV-2 contains the spike (S), matrix (M), and envelope (E) proteins. Protein S in particular is known to play a crucial role in viral entry into host cells and infectivity and is a critical target for inducing antibodies, especially NAbs against SARS-CoV-2 (11). The viral core contains the nucleocapsid (N) protein; since protein N is “shielded” by viral membranes, anti-N Abs are less likely to directly neutralize SARS-CoV-2 (12).

The major gene-based vaccines that have been developed thus far solely target protein S, the viral ligand to bind angiotensin-converting enzyme 2 (ACE2) on host cells, thus preventing the viral entry into the host cells. Many clinical studies have reported the humoral response to these COVID-19 vaccinations. A common observation is that messenger RNA (mRNA) vaccines (RVs) such as BNT162b2 (Pfizer BioNTech) have higher immunogenicity and neutralization efficacy against S protein, measured via anti-S Abs and NAbs, than whole-virus inactivated vaccines (IVs) such as BBIBP-CorV (Sinopharm) (13). However, studies also revealed that IVs, like RVs, are effective in reducing the severity and mortality of SARS-CoV-2 (14, 15). This raises the question of whether the ability of IVs to induce Abs against the other viral proteins of SARS-CoV-2, *e.g.*, protein N compensates for its rather low titer of anti-S Abs and NAbs. Indeed, anti-N Ab was detected in convalescents (16) and was shown to improve viral clearance (17).

The Macao Special Administrative Region of China possesses a small population of around 670,000 in a strictly bordered territory. It is one of the first places that took non-pharmaceutical interventions to control its local outbreak of COVID-19 since early 2020, keeping at less than hundred infections (including non-symptomatic cases) until the first larger increase (to near two thousand) on 18 June 2022 (18). Macao also has a solid immunization policy with two to three doses of BBIBP-CorV and/or BNT162b2 vaccines before its first local spread of SARS-CoV-2 (18). Therefore, it is a region with very clear background of infection and vaccination compared to many other regions or countries that have suffered high infections. To this end, we decided to study how both vaccine types synergize in inducing the humoral immune responses. We recruited 147 participants in Macao from April 2021 to August 2022 and collected their serum to study the effects of BBIBP-CorV and BNT162b2 vaccines. Our findings demonstrate that BBIBP-CorV and BNT162b2 synergize to induce anti-S and -N Abs and NAb to SARS-CoV-2 which otherwise cannot be achieved by either vaccine type alone.

## Methods

### Ethics statement

This work was conducted under an ethics protocol of the University of Macau (#BSERE21-APP010-FHS). Only human subjects above age of 18, excluding pregnant women, prisoners, and mentally/cognitively impaired individuals, were recruited following informed consent. Ten ml of peripheral blood was drawn from each participant for antibody detection. Identifiable information of donors such as names, telephone #s, ID #, and addresses was removed from the samples and stored in a secured computer in the principal investigator’s office.

### Cohort definition and serum collection

A total of 147 individuals were recruited in this study including 11 convalescents and were divided into several groups according to their vaccination status and types. 2IV: individuals who received two doses (0.5 mL) of BBIBP-CorV (IV); 3IV: individuals who received three doses of BBIBP-CorV; 2RV: individuals who received two doses (0.6 mL) of BNT162b2 (RV); 3RV: individuals who received three doses of BNT162b2; 2IV+1RV: individuals who received two doses of BBIBP-CorV and one booster of BNT162b2. Whole blood (10 mL) was drawn from the individuals at Kiang Wu hospital and divided into two parts: the first part was mixed with the anticoagulant heparin for blood cell counting, and the second part was not mixed with the anticoagulant for serum collection. Serum was isolated after centrifugation and stored at -80°C for Ab measurements and biochemical testing.

### Serologic assays

The quantitative detection of serum antibody for SARS-CoV-2 S (spike, RBD), N (nucleocapsid) and the neutralizing antibody (NAb) was performed via electrochemiluminescence immunoassay with Elecsys® Anti-SARS-CoV-2 S kit (Roche), Elecsys® Anti-SARS-CoV-2 kit (N antigen as target, Roche), and MAGLUMI® SARS-CoV-2 Neutralizing Antibody kit (Snibe), respectively, according to the manufacturer’s instructions. All results were automatically analyzed and generated in U/mL, cut-off index (COI) or μg/mL based on the manuals. For anti-SARS-CoV-2 S, < 0.80 U/mL was interpreted as negative and ≥ 0.80 U/mL as positive. For anti-SARS-CoV-2 (N), COI < 1.0 was interpreted as non-reactive and COI ≥ 1.0 as reactive. For Nab, ≥ 0.3 μg/mL was interpreted as reactive.

### Cytokine measurements

Serum concentrations of IFN-γ, TNF-α, and IL-2 were measured through enzyme-linked immunosorbent assay (ELISA) using kits from Shanghai Kechen MicroBioTech, Ltd., (cat. # KCW301152, KCW320663, and KCW320226, respectively) according to the manufacturer’s instructions. Concentrations were calculated based on standard curves established using provided standard samples and the sensitivity of these assays was 1.0 pg/mL.

### Statistical analysis

All analyses were performed using a Bayesian framework for hypothesis testing (19). For the hypothesis testing, the likelihood function of the data under a logarithmic (with base 10) transformation of a group was assumed to follow a Student-t distribution. The priors of the model were assumed to be an uninformative normal prior for the mean for all groups, with its hyperparameters to be the pooled empirical mean and five-fold of the pooled empirical standard deviation of the whole data set; the standard deviation for all groups follows a uniform prior; the prior for the degree of normality exponentially distributed with a mean of 30. The posteriors were sampled using a Markov chain Monte Carlo (MCMC) sampling scheme through a probabilistic programming framework in Python, PyMC3 (20), with a No-U-Turn sampler (NUTS) (21).

## Results

**Figure 1:**
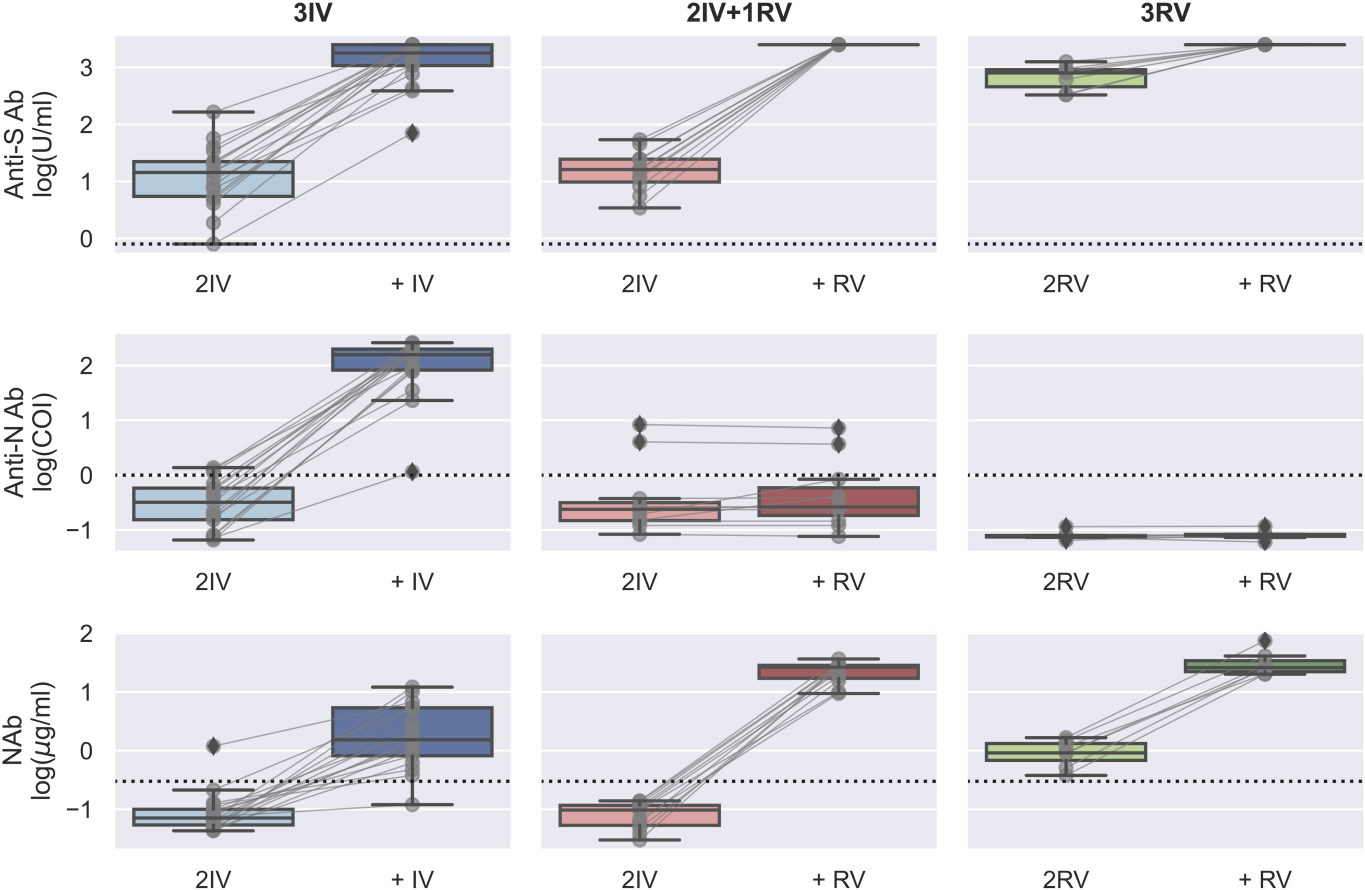
Serologic assays against SARS-CoV-2 for the booster effect of the third vaccinations. Abs against proteins S (top) and N (middle) and Nab (bottom) were determined on serum samples collected before and after the (third) booster dose with either BBIBP-CorV (IV) or BNT162b2 (RV). *N* = 16, 8, and 7 biologically independent samples for 3IV (blue), 2IV+1RV (red), and 3RV (green), respectively. Each pair of connected grey points represents a participant. Each dashed line represents the reactive level for the corresponding test (see Methods). A Bayesian analysis of the booster effects is shown in **Figure S1**.

**Figure 2:**
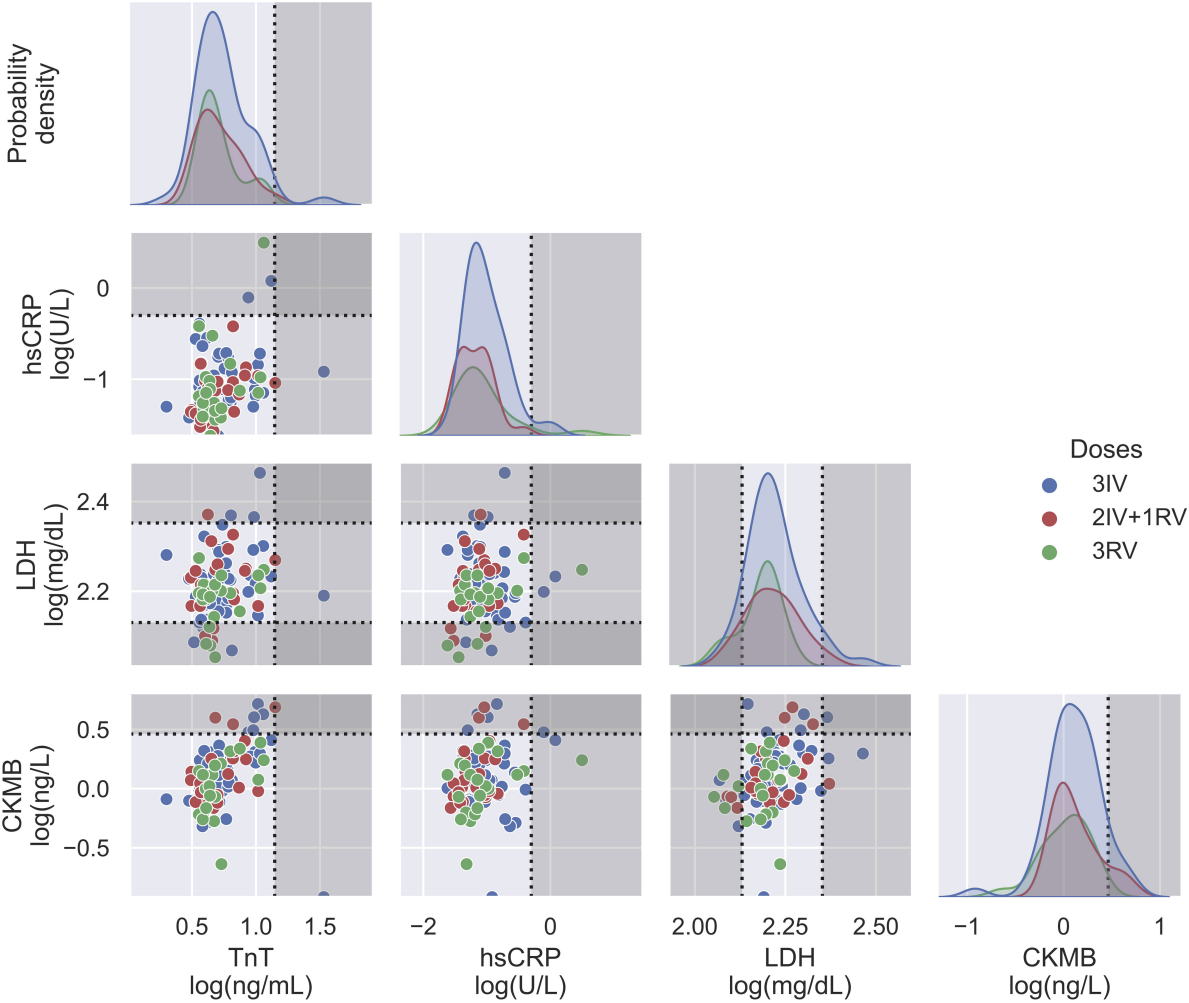
Biochemical assays on serum from three vaccination groups. Some relevant indices including TnT, hsCRP, LDH, and CKMB were displayed in both probability density plots (diagonal panels) and pairwise scatterplots (off diagonal panels), showing no major differences between the vaccination groups. *N* = 50, 24, and 21 for 3IV, 2IV+1RV, and 3RV, respectively. Each point in the pairwise scatterplot represents a participant. Dashed lines represent the reference level for the corresponding index, where the non-grayed areas are the normal ranges.

Using the serologic assays for anti-S and -N Abs and NAb to SARS-CoV-2 in the participants with vaccinations, we first analyzed data from those tested the Abs before and after the booster dose (**Figure 1**). The IV booster (third) significantly increased the level of anti-N Ab (3IV participants) when comparing to the groups with the RV booster (2IV+1RV and 3RV participants). However, after administration of the RV booster, the levels of anti-S Ab and NAb markedly increased, compared to the IV booster (**Figure S1**). These data show that the booster dose with RV enhanced anti-S Ab and NAb but not anti-N Ab regardless of the vaccine types (IV or RV) in the previous vaccinations, whereas the booster dose with IV promoted anti-N Ab only. Most individuals showed a normal range of blood cell counts (data not shown) and biochemical indices in the serum such as high-sensitivity C-reactive protein (hsCRP, a parameter for inflammation), lactate dehydrogenerase (LDH, a general marker for tissue damage), high-sensitivity troponin-T (TnT, a specific marker for cardiac damage), and creatine kinase MB (CKMB, a specific marker for muscle damage) (**Figure 2**), indicating no obvious toxicity of the vaccines, consistent with the literature (22, 23).

**Figure 3:**
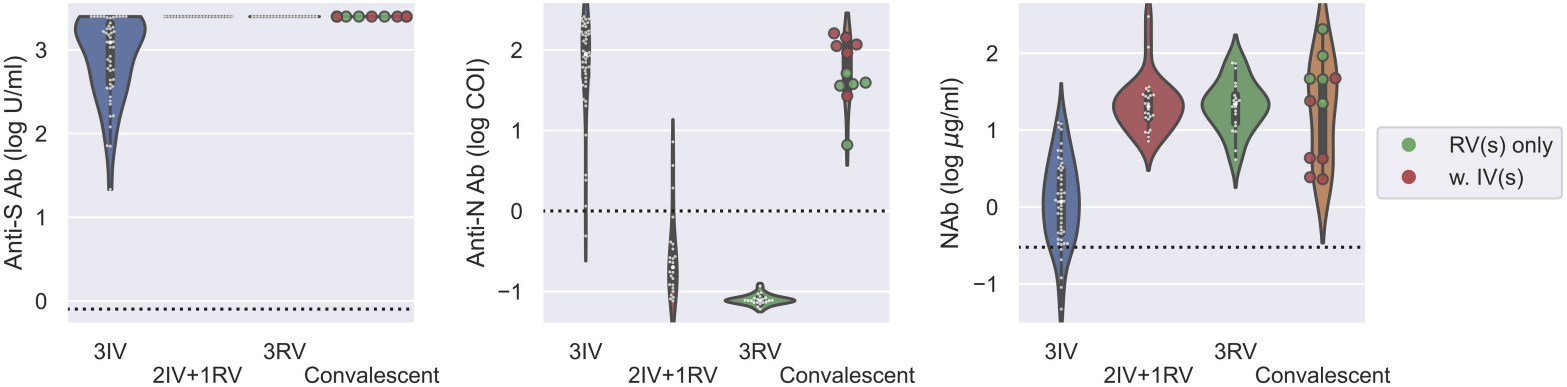
Serologic assays against SARS-CoV-2 for comparison of vaccinees and convalescents. Abs against proteins S (left) and N (middle) and Nab (right) were determined on serum samples collected from various groups of vaccinees as well as for convalescents within two months after cure. *N* = 50, 24, 21, 11 for 3IV, 2IV+1RV, 3RV, and convalescents, respectively. Each point represents a participant. The vaccination history of the convalescents was marked with green (*N* = 5) for convalescents with RV(s) and red (*N* = 6) for convalescents with IV(s). The dashed line in each panel represents the reactive level for the corresponding test.

Next, we analyzed all the data from those tested the Abs with the booster dose as well as the convalescents. Using the same serologic assays for 11 convalescents within two months after cure, we found that the convalescents had similar levels of anti-S Ab and NAb as the 2IV+1RV and 3RV vaccinees (**Figure 3**). The level of anti-N Ab for the convalescents was like that for 3IV but higher than that for 2IV+1RV, and the latter was higher than that for 3RV (**Figure S2**). Convalescents who received RV vaccination(s) (*N* = 5) had a similar level of anti-N Ab to that of the healthy 3IV vaccinees, and the levels in both groups were higher than that in the healthy 3RV vaccinees (**Figure 3**). These data suggest that COVID-19 infections promoted not only the anti-S Ab and NAb levels but also the anti-N Ab level regardless of the vaccination history. To test cellular response to the vaccinations, we measured IFN-γ, TNF-α, and IL-2 related to T-cell activation before and after the boosters. No significant differences in the basal levels of these cytokines in the serum of the participants were observed among the various vaccination groups (**Figure S4**).

## Discussion

In this study, we compared the levels of anti-S, anti-N Ab, and NAb in vaccinees in Macao following the use of IV, RV, or their heterologous use. We found that the RV booster following 2IV or 2RV increased anti-S Ab and NAb but not anti-N Ab. In contrast, only the IV booster increased anti-N Ab, consistent with the nature of IV which theoretically contained most, if not all, of the viral proteins including protein N, although IV induced lower anti-S Ab and NAb levels than RV. Our results highlight the benefit of IV to induce humoral response to all the viral proteins.

A recent study in Hong Kong reported that BNT162B2 (3 doses, 3RV) was more effective than CoronaVac (3 doses, 3IV) to reduce the rate of mild/moderate disease of Omicron BA.2, whereas both were similarly effective (over 90%) in reducing severe/fatal disease and mortality (14). Vaccinations with IVs alone prevented the infection of Omicron by only 13.2% but reduced its severe/fatal disease by 88.6% and mortality by 91.7% (15). These findings have demonstrated the effectiveness of IV, like RV, in preventing the severity of SARS-CoV-2 variants such as Omicron.

Anti-S Ab and NAb are crucial for prevention of SARS-CoV-2 infection by blocking the viral entry into host cells. However, IV-induced Abs against multiple viral proteins may contribute to the reduction of the severity and mortality, since the other viral proteins are also critical for the pathological development of COVID-19 (24). Furthermore, a study showed that around 7% of Abs isolated from 3IV vaccinees were effective against Omicron (25), indicating that these Abs may contribute to the effectiveness of 3IV in reducing the rate of severe/fatal disease by targeting the viral proteins, including protein N. To survive the host immune resistance developed via infections or vaccinations, SARS-CoV-2 has countered via frequently developed mutations in its S-encoding gene. All the variants of SARS-CoV-2 identified thus far contain much more frequent mutations in protein S than the other proteins, *e.g.*, protein N (26, 27). It has been reported that, compared to RVs, IVs induced higher T-cell responses against multiple SARS-CoV-2 proteins (28, 29), suggesting the potential advantage of IVs against highly mutated SARS-CoV-2. However, we didn’t observe significant differences in the seral levels of cytokines related to T-cell activation among the various vaccination groups (**Figure S4**). Such differences might only occur shortly after antigen reattack such as a booster vaccination or infection and would gradually disappear with time.

Currently, even 4RV could not prevent Omicron infection, and the viral load and infectivity increased in infected people following 4RV (30). Yet, the NAb activity with 2IV+1RV reached almost as high as that in convalescent with 1RV but higher than that in healthy vaccinees with 1RV (31), indicating the effectiveness of IV resembling the immune response following an infection. In a study of over 14 million people in Brazil, 2IV+1RV reduced infection and severe disease by 92.7% and 97.3%, respectively, while 2IV alone reduced infection and severe disease by 55.0% and 82.1%, respectively (32). These results further support the beneficial effect of heterologous vaccinations with the two vaccine platforms and are consistent with our findings.

In summary, we found that heterologous vaccinations of IV and RV induce not only a high titer of anti-S Ab and NAb but also anti-N Ab. Compared to the single-target effect of RV, the heterologous vaccinations of IV and RV launches a multi-target attack to the virus during not only the viral entry but also the subsequent pathological processes. This may be correlated to the low infection rate and reduced severity and mortality in those with the heterologous vaccinations as discussed above. Further research is necessary to study the duration of the vaccination effects and the efficacy of anti-N Ab in interfering with the pathological process of SARS-CoV-2 and protecting against the viral variants. Nevertheless, our results provide new evidence for the benefit of heterologous vaccinations of IV and RV from a local population contained under stringent conditions, which shall support the decision-making for future vaccine development and vaccination strategies.

## Data availability statement

The original contributions presented in the study are included in the article/**Supplementary Material**. Further inquiries can be directed to the corresponding authors.

## Ethics statement

The studies involving human participants were reviewed and approved by the University of Macau (#BSERE21-APP010-FHS). The patients/participants provided their written informed consent to participate in this study.

## Author contributions

R-HX, HMN, and CLoL conceived of and designed the research. HMN, SF, EL, XJT, SL, CN, NC, KL and CLeL performed the experiments, and CLoL analyzed the data. CLoL and R-HX wrote the manuscript. R-HX gave the final approval of the manuscript. All authors contributed to the article and approved the submitted version.
