# Corrigendum: Heterologous vaccination with inactivated vaccine and mRNA vaccine augments antibodies against both spike and nucleocapsid proteins of SARS-CoV-2: a local study in Macao

**DOI:** 10.3389/fimmu.2024.1375028

**Published:** 2024-02-06

**Authors:** Hoi Man Ng, Chon Lok Lei, Siyi Fu, Enqin Li, Sek In Leong, Chu Iong Nip, Nga Man Choi, Kai Seng Lai, Xi Jun Tang, Chon Leng Lei, Ren-He Xu

**Affiliations:** ^1^ Laboratory Department, Kiang Wu Hospital, Macao, Macao SAR, China; ^2^ Faculty of Health Sciences, University of Macau, Macao, Macao SAR, China; ^3^ Laboratory Department, Zhuhai Hospital of Integrated Traditional Chinese and Western Medicine, Zhuhai, China

**Keywords:** SARS-CoV-2, inactivated vaccine, mRNA vaccine, heterologous vaccinations, antibodies, spike proteins, nucleocapsid proteins, Macao

In the published article, there was an error in affiliation 3. Instead of “Laboratory Department, Zhuhai Hospital of Combined Traditional Chinese and Western Medicine, Zhuhai, China”, it should be “Laboratory Department, Zhuhai Hospital of Integrated Traditional Chinese and Western Medicine, Zhuhai, China”.

The authors apologize for this error and state that this does not change the scientific conclusions of the article in any way. The original article has been updated.

