# Supplementary material for: Heterologous vaccination with inactivated vaccine and mRNA vaccine augments antibodies against both spike and nucleocapsid proteins of SARS-CoV-2: a local study in Macao
Source: Front Immunol. 2023 May 12;14:1131985. doi: 10.3389/fimmu.2023.1131985 (PMC10213252; doi:10.3389/fimmu.2023.1131985)
Supplement: Supplementary file 1 [file Image_1.pdf]

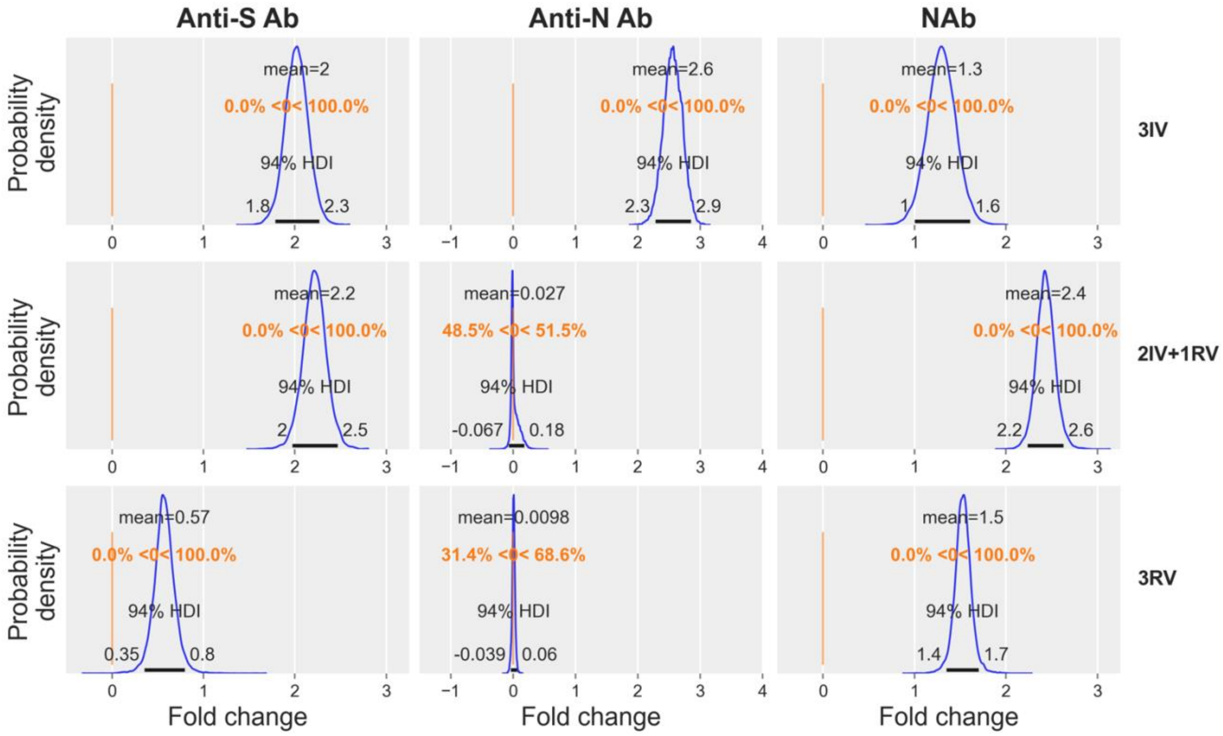

Figure S1. Bayesian analysis for the mean differences of serologic assays for the booster effect of the third vaccination. Displayed are the log fold change of Abs against proteins S (left) and N (middle) and Nab (right) in serum samples collected before and after the booster dose. The probabilities of which there is a difference in the log-transformed mean estimate (fold change of 10) are shown. Here, *N* is the same as Figure 1. All the fold changes indicate an increase in the Ab levels to various degrees after the booster, except that there is no change in the anti-N Ab level (close to the orange vertical line) after receiving the RV booster (middle and bottom rows). Note that the baseline for 3RV (the last row) is different from the other two groups, therefore the comparison between 3IV and 2IV+1RV (the top and middle rows) may better reveal the differences in the booster effect. The mean value marks the mean folder change, the range marked in orange refers to the distribution with reference to no change, and the highest-density interval (HDI) indicates the most credible region of the corresponding analysis.
