# Supplementary material for: Heterologous vaccination with inactivated vaccine and mRNA vaccine augments antibodies against both spike and nucleocapsid proteins of SARS-CoV-2: a local study in Macao
Source: Front Immunol. 2023 May 12;14:1131985. doi: 10.3389/fimmu.2023.1131985 (PMC10213252; doi:10.3389/fimmu.2023.1131985)
Supplement: Supplementary file 2 [file Image_2.pdf]

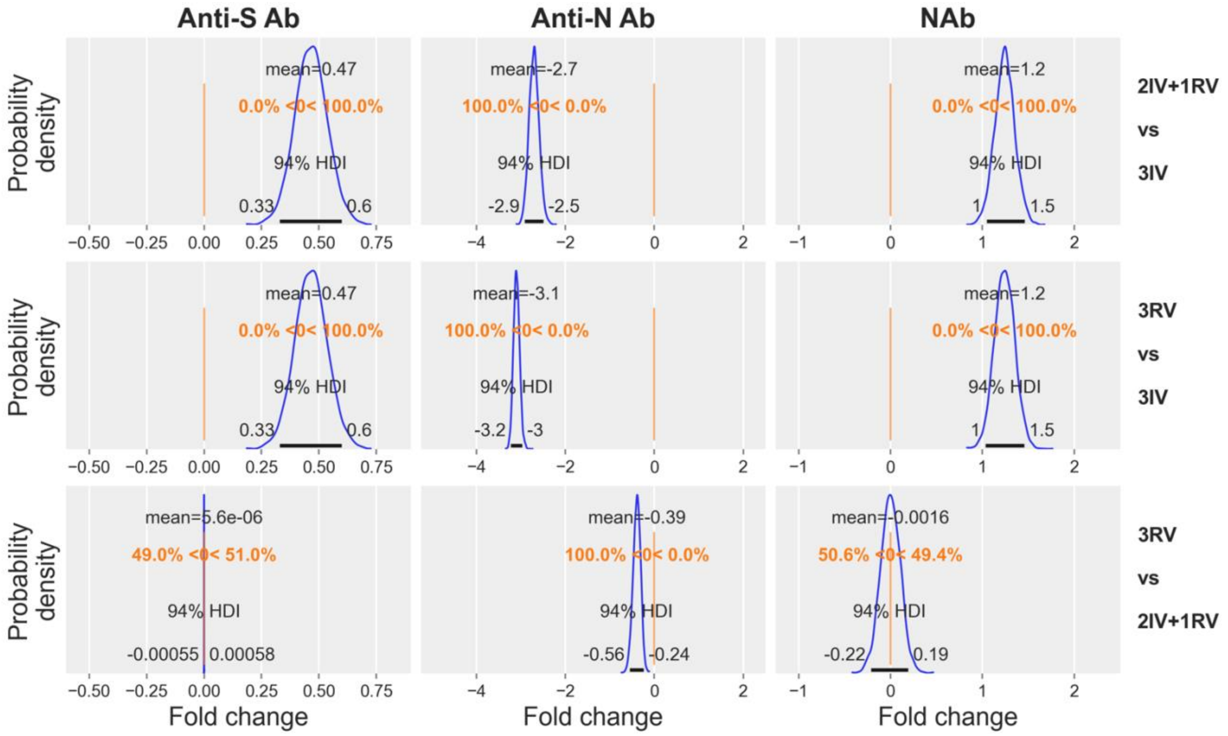

Figure S2. Bayesian analysis for the mean differences of serologic assays between various combinations of vaccinations. Displayed are the log fold change of Abs against proteins S (left) and N (middle) and NAb (right) in the serum samples collected from the various vaccination groups. The probabilities of which there is a difference in the log-transformed mean estimate (fold change of 10) are shown. N = 50, 24, 21 for 3IV (top), 2IV+1RV (middle), and 3RV (bottom), respectively. The mean, range, and HDI are labeled as for Fig. S1.
