# Supplementary material for: Heterologous vaccination with inactivated vaccine and mRNA vaccine augments antibodies against both spike and nucleocapsid proteins of SARS-CoV-2: a local study in Macao
Source: Front Immunol. 2023 May 12;14:1131985. doi: 10.3389/fimmu.2023.1131985 (PMC10213252; doi:10.3389/fimmu.2023.1131985)
Supplement: Supplementary file 3 [file Image_3.pdf]

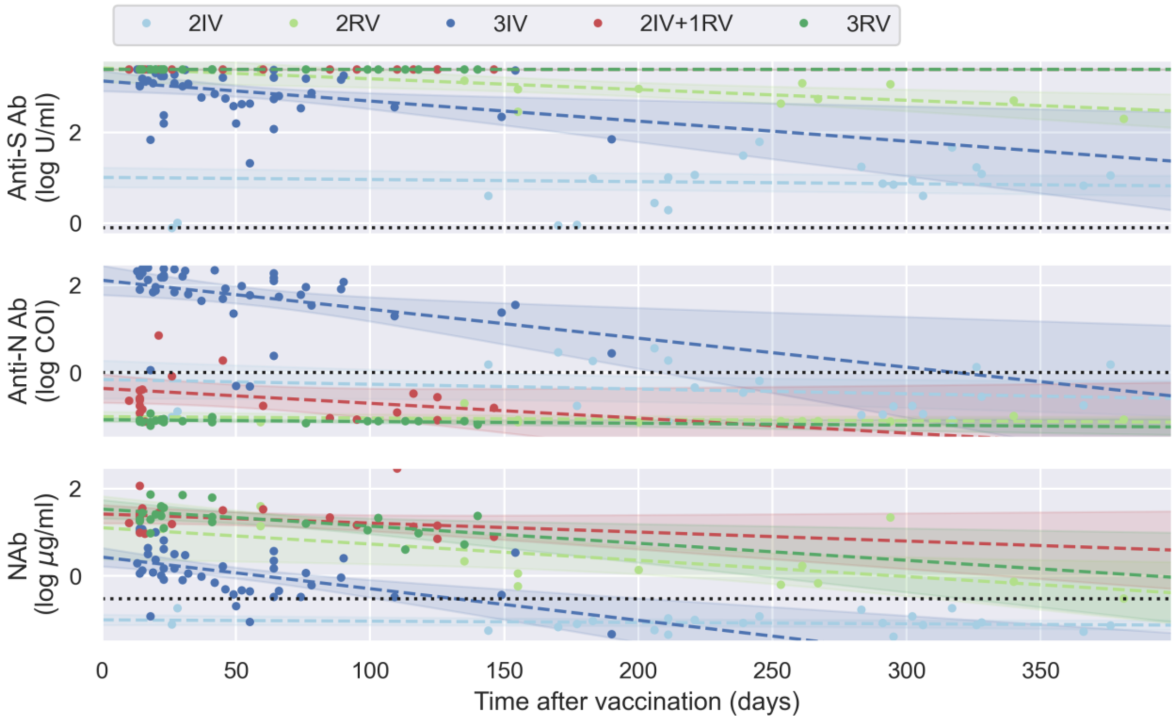

Figure S3. The relationship between vaccination-serum collection time interval and the various antibody responses. Abs against proteins S (top) and N (middle) and NAb (bottom) were plotted over time after vaccination. Here, N = 22, 13, 50, 24, 21 for 2IV, 2RV, 3IV, 2IV+1RV, and 3RV, respectively. Each point represents a participant. Dashed lines represent the reactive level for the corresponding test. A Bayesian linear regression model with additive white noise was used for analyzing the transformed response level-time relationship, assuming a normal prior for the coefficient, and intercept, with mean zero and standard deviation of 0.01, and 5, respectively, and a half-normal prior with standard deviation of 1 for the white noise. The posteriors were sampled according to the procedure described in Methods. The mean trends of the durability of 2IV and 2RV responses are similar, whilst the responses of the booster doses with much higher response levels decay much quicker than 2IV and 2RV.
