# Supplementary material for: Heterologous vaccination with inactivated vaccine and mRNA vaccine augments antibodies against both spike and nucleocapsid proteins of SARS-CoV-2: a local study in Macao
Source: Front Immunol. 2023 May 12;14:1131985. doi: 10.3389/fimmu.2023.1131985 (PMC10213252; doi:10.3389/fimmu.2023.1131985)
Supplement: Supplementary file 4 [file Image_4.pdf]

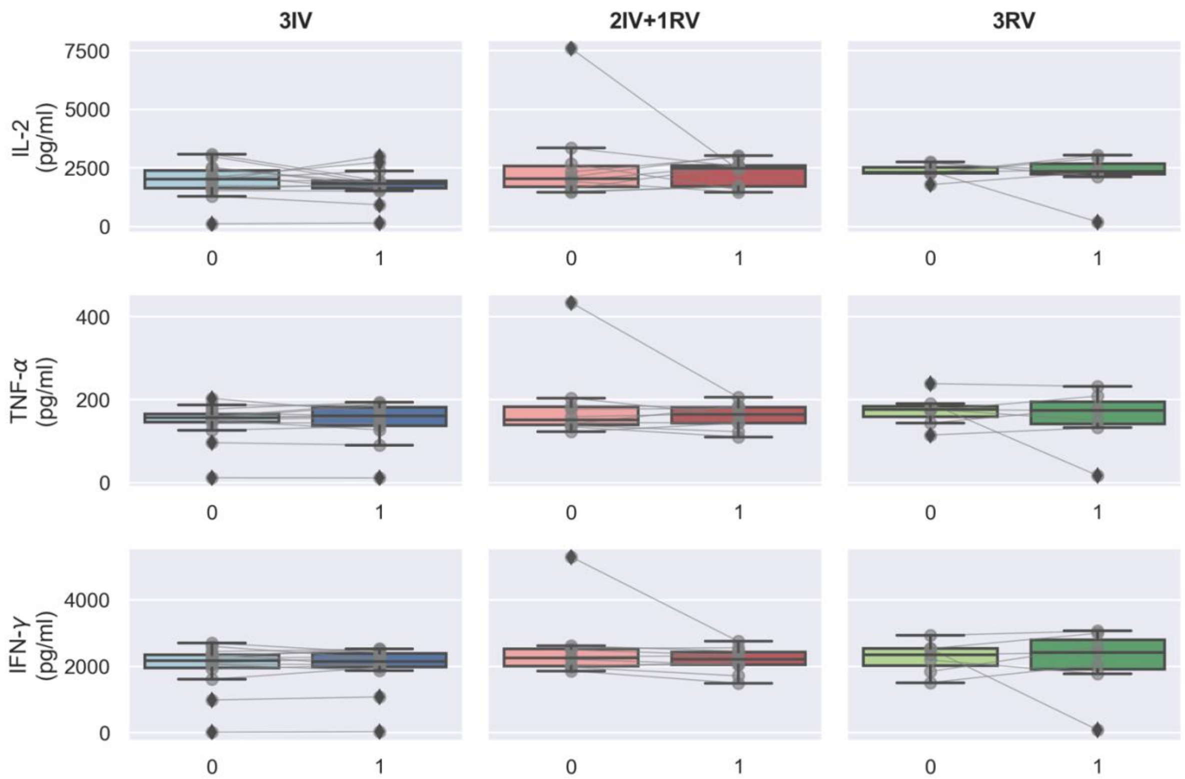

Figure S4. Cytokines related to T-cell activation. IL-2, TNF-α, and IFN-γ were determined on serum samples collected before and after the booster (third) dose with either IV or RV. N = 16, 8, and 7 biologically independent samples for 3IV (blue), 2IV+1RV (red), and 3RV (green), respectively. Each pair of connected grey points represents a participant.
